# Factors Associated with PMTCT Cascade Completion in Four African Countries

**DOI:** 10.1155/2016/2403936

**Published:** 2016-10-31

**Authors:** Jodie Dionne-Odom, Thomas K. Welty, Andrew O. Westfall, Benjamin H. Chi, Didier Koumavi Ekouevi, Margaret Kasaro, Pius M. Tih, Alan T. N. Tita

**Affiliations:** ^1^University of Alabama at Birmingham, Birmingham, AL, USA; ^2^Cameroon Baptist Convention Health Services (CBCHS), Bamenda, Cameroon; ^3^University of North Carolina at Chapel Hill, Chapel Hill, NC, USA; ^4^Research Center INSERM U1219, ISPED, Université de Bordeaux, Bordeaux, France; ^5^PAC-CI Program, ANRS Site, Abidjan, Côte d'Ivoire

## Abstract

*Background. *Many countries are working to reduce or eliminate mother-to-child transmission (MTCT) of HIV. Prevention efforts have been conceptualized as steps in a cascade but cascade completion rates during and after pregnancy are low.* Methods. *A cross-sectional survey was performed across 26 communities in Cameroon, Cote d'Ivoire, South Africa, and Zambia. Women who reported a pregnancy within two years were enrolled. Participant responses were used to construct the PMTCT cascade with all of the following steps required for completion: at least one antenatal visit, HIV testing performed, HIV testing result received, initiation of maternal prophylaxis, and initiation of infant prophylaxis. Factors associated with cascade completion were identified using multivariable logistic regression modeling.* Results. *Of 976 HIV-infected women, only 355 (36.4%) completed the PMTCT cascade. Although most women (69.2%) did not know their partner's HIV status; awareness of partner HIV status was associated with cascade completion (aOR 1.4, 95% CI 1.01–2.0). Completion was also associated with receiving an HIV diagnosis prior to pregnancy compared with HIV diagnosis during or after pregnancy (aOR 14.1, 95% CI 5.2–38.6).* Conclusions. *Pregnant women with HIV infection in Africa who were aware of their partner's HIV status and who were diagnosed with HIV before pregnancy were more likely to complete the PMTCT cascade.

## 1. Background

The worldwide elimination of mother-to-child transmission of HIV is an ambitious goal. Despite significant progress toward this goal over the past decade, most of the 220,000 new pediatric HIV infections in 2014 were attributed to vertical transmission [1]. Successful prevention of mother-to-child HIV transmission (PMTCT) requires early HIV diagnosis, consistent access to antiretroviral (ART) medications, and engagement in care during and after pregnancy [2]. Current international guidelines recommend lifelong ART for all pregnant women (PMTCT World Health Organization (WHO) Option B+) and highlight the importance of retention in care to improve maternal and child health [3, 4]. The HIV PMTCT cascade provides an effective tool to visualize prevention efforts in pregnant women and to identify opportunities for improvement as highlighted by the landmark PEARL study (PMTCT Effectiveness in Africa: Research and Linkages to Care) which took place in four African countries. The PEARL study had two components: a community-based component with household-level surveys and a facility-based component with the collection of cord blood to measure nevirapine levels. It was designed to measure infant HIV-free survival at the population level. It also estimated the effectiveness of PMTCT programs by comparing HIV pregnancy outcomes in Sub-Saharan Africa collected from women at the facility level compared to women surveyed in the “real world” community setting. This study defined the PMTCT cascade as five essential and sequential steps: at least one antenatal care (ANC) visit, an HIV test performed, the HIV test result received, initiation of maternal ART prophylaxis, and initiation of infant ART prophylaxis [5]. Cascade completion rates are suboptimal among HIV-infected women during and after pregnancy [6–9]. The completion rate in the facility component of the PEARL study across all sites was 51% and in another study in South Africa, 58% of HIV-infected pregnant women in care were lost to follow-up (LFU) by 6 months after delivery [5, 10]. Certain factors have been associated with LFU: younger age (<25 years old), ART initiation during pregnancy, and pregnancy itself [11–14]. On the other hand, improved PMTCT cascade completion rates have been associated with male partner involvement, couples HIV counseling and testing programs, and HIV status disclosure [15–18]. Understanding the predictors of cascade completion can be used to improve PMTCT programs and maternal and infant HIV-associated outcomes.

In a recent analysis of the PEARL community-level data by Chi et al., the cascade completion rate was lower (36.4%) than the rate calculated with facility level data (51%) although both were suboptimal despite high rates of access to ANC care (97.3%) and facility deliveries (93.2%) [19]. For this analysis, we used data from the community-based component of the PEARL study and sought to identify individual characteristics associated with PMTCT cascade completion.

## 2. Methods

### 2.1. Study Design

The PEARL study was conducted in Cameroon, Cote d'Ivoire, South Africa, and Zambia during 2007–2009. The 165-question community-based household survey was administered by trained study teams. Households in chosen PMTCT program catchment areas were randomly selected for participation and blood was collected from mother-infant pairs for HIV testing [19]. Women who reported a birth in the past 24 months were eligible to participate and the PMTCT cascade was recreated for women with HIV infection based on their responses to survey questions. Standard of care for PMTCT medications varied by country, but, overall, 36% of women received prophylaxis with antenatal zidovudine and peripartum nevirapine, 37% received peripartum nevirapine only, 18% received combination therapy (defined as receiving more than one antiretroviral drug to treat HIV), and 76.3% of infants received prophylaxis with zidovudine and nevirapine.

### 2.2. Study Outcome

Successful completion of the PMTCT cascade was the main study outcome and it required completion of five steps. The five-step cascade was defined as follows: having had at least one ANC visit, having an HIV test performed, receipt of the HIV test result, initiation of maternal antiretroviral prophylaxis, and initiation of infant antiretroviral prophylaxis.

### 2.3. Independent Variables

Available sociodemographic and healthcare-related variables were examined for potential association with cascade completion. Sociodemographic variables included age, marital status, education, parity, household size (total number of persons), source of household water, ownership of a mode of transportation (bicycle, motorcycle, or car), and current employment outside the home. Country of origin was not included as a variable since it was a stratification factor in the analysis.

Healthcare-related variables included timing of HIV diagnosis (before, during, or after last pregnancy), partner HIV testing and status (with separate multivariable models created for HIV status known/unknown and HIV test positive/negative/unknown), method used (ever) for family planning (modern, traditional, or none), gestational age at initial antenatal care visit (self-report), ANC location (hospital, health center, health post, or other), and ANC facility type (public facility, faith-based organizational facility, or other). The family planning variable was categorized into modern or traditional methods using standard definitions. Modern methods were defined according to the WHO definition as sterilization, intrauterine device (IUD), implants, injectables, lactational amenorrhea, pill, or condoms. Pill and condoms were grouped as short-acting modern methods. Traditional methods were defined as natural family planning or withdrawal. Information about HIV status disclosure by participants was not available.

### 2.4. Data Analysis

Baseline characteristics of women were compiled with percentages for categorical variables. The denominator used for the cascade was the 976 women identified with HIV infection with complete data from the community survey. Univariate and multivariable (MV) logistic regression analysis was used to create a model with the outcome of successful cascade completion. Variables were chosen for inclusion in the final MV analysis based on significance in the univariate model (*p* < 0.05) and factors associated with cascade completion in prior studies (age, marital status, education, socioeconomic status, and timing of ANC entry) regardless of univariate significance [8]. The model was stratified by country with a fixed effects conditional logistic regression approach where each stratus was allowed its own intercept. The sample size was not sufficient for analysis at the individual country level. All analyses were performed with SAS 9.4 (Cary, NC).

### 2.5. Ethics

The primary study was approved by the institutional review boards of the University of Alabama at Birmingham (UAB), US Centers for Disease Control (CDC), and local research ethics committees in Cameroon, Zambia, South Africa, and Cote d'Ivoire. A waiver was obtained from the Cameroon Baptist Convention Health Services (CBCHS) IRB to perform this additional analysis using a deidentified, released dataset.

## 3. Results

**Figure 1:**
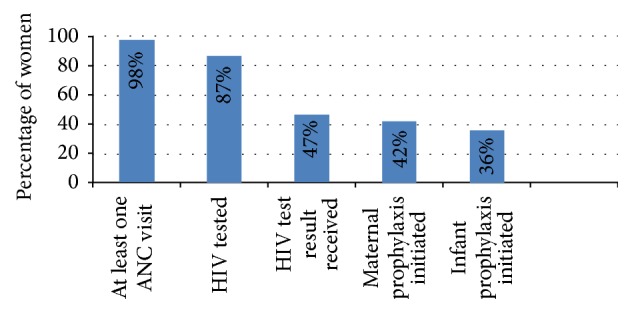
PEARL PMTCT cascade among 976 HIV-infected pregnant women in four African countries (data from Chi et al. [19]).

From April 2007 to March 2009, 7985 mother-infant pairs were enrolled in the four-country household-level community-based component of the PEARL study and 1014 women (12.7%) tested positive for HIV. Complete data available for 976 HIV-infected women comprised our study population. In this group, 355 (36.4%) completed every step of the cascade (Figure 1). As reported previously by Chi et al., in relation to the study population as a whole, 98% of women attended at least 1 ANC visit, 87% were tested for HIV, 47% received a positive HIV test result, 42% initiated maternal prophylaxis, and 36% initiated infant prophylaxis [19]. The most significant dropout was among women who reported that they were tested for HIV but did not receive the results.

The characteristics of women who completed the cascade versus those who did not are shown in Table 1. Overall, the median age of the cohort was 27 and most participants were from Zambia (45.5%) or South Africa (37.4%). Most women were married (56%), had attended at least secondary school (55.9%), and had never used birth control (43.6%), and the median parity was 2. A majority did not own a method of transportation (75.7%) and nearly all women reported attending antenatal care at least once. Most women presented for ANC care in the 2nd trimester (67.9%) at a governmental health center clinic and nearly 1 in 4 women were diagnosed with HIV during the most recent pregnancy. Nearly 70% of women did not know the HIV status of their male partner. In general, sociodemographic characteristics did not appear to differ by cascade completion status whereas several health-related factors including the timing of HIV diagnosis, partner HIV testing, use of birth control, ANC location, and facility type were significantly different (Table 1).

In the univariate analysis, the strongest association with cascade completion was in women diagnosed with HIV prior to pregnancy compared with HIV diagnosis during or after pregnancy (OR 12.3, 95% CI 5.3–28.6) (Table 2). Other factors associated with cascade completion included age above 30, parity of 4, household size above 3, and use of long-acting modern birth control methods. Maternal employment, household water source, and ownership of a television or cellphone were included in the model but were not associated with cascade completion. Due to collinearity between ANC location and facility type, only facility type was included in the MV model.

The MV analysis based on 843 observations (Table 2) shows a significant association between cascade completion and awareness of partner HIV status (aOR 1.4, 95% CI 1.01–2.0), HIV diagnosis prior to pregnancy (aOR 14.1, 95% CI 5.2–38.6), and use (ever) of a long-acting method of contraception (aOR 2.0, 95% CI 1.3–3.0). When the multivariable analysis was repeated using a three-level variable for partner HIV status (negative/positive/unknown), having an HIV-positive partner was associated with cascade completion compared to having an HIV-uninfected partner (OR 6.1, 95% CI 3.0–12.3). In this model, other findings were similar except for an association of cascade completion with household size of 4 persons (compared to 1-2) with an OR 2.9, 95% CI 1.02–8.18 and a lack of association with owning a means of transportation or having ANC at a facility that was run neither by the government nor an FBO.

## 4. Discussion

The PMTCT cascade completion rate from entry into ANC care to receipt of infant antiretroviral prophylaxis was only 36.4% using community-based survey data. This rate is comparable to more recent studies in Africa showing cascade completion rates ranging from 11 to 70% [20–23]. Fortunately, access to HIV testing and antiretroviral therapy in pregnancy is improving as PMTCT programs are upscaled worldwide [3]. Antenatal HIV testing is not yet universal but 77% of pregnant women with known HIV infection had access to antiretrovirals in 2015 [24]. This number varies by region with 90% ART coverage for pregnant women living with HIV in East/South Africa but only 48% coverage among pregnant women in West/Central Africa. These numbers also underestimate the need for ART since many women are unaware of their infection. Although PMTCT can be incorporated into routine antenatal care, studies now show that many HIV-infected women who are engaged in care during pregnancy are lost to follow-up during the postpartum period [6, 25–28]. This creates an important barrier to maintaining virologic suppression in women with multiple births and more effective PMTCT programs are needed to facilitate long-term retention in care and reach the ambitious UN AIDS target of 90-90-90 by 2030.

Our study is the first to our knowledge to document the importance of awareness of partner HIV status in terms of PMTCT cascade completion. Nearly 70% of women with HIV were not aware of their partner's HIV status in our study and 41% of women with a recent pregnancy in South Africa were similarly unaware [29]. Whether this beneficial effect is mediated via a reduction in stigma or an increase in support is not clear but cascade completion rates were highest among women with concordant, HIV-infected male partners. The importance of male partner involvement with the PMTCT process has recently been recognized and women surveyed have been strongly supportive of this concept [30–32]. Programs offering couples voluntary counseling and testing in the antenatal clinic setting have shown mixed efficacy, with improved infant nevirapine use in Kenya but not in Rwanda or Zambia [16, 33, 34]. Awareness of partner HIV status has also been associated with other beneficial outcomes among women with HIV, such as higher facility delivery rates [35]. In Kenya, HIV-infected pregnant women who reported that their partner had been tested for HIV had lower rates of vertical transmission and infant mortality, but awareness of partner HIV status was not captured [36]. Current interventions to improve PMTCT partner testing include mailed invitations and home-based HIV testing procedures [37–41]. Although our study did not capture disclosure information, HIV status disclosure during pregnancy (to anyone, but particularly to the male partner) has been associated with ARV use in PMTCT [15, 42, 43].

HIV diagnosis prior to pregnancy was also independently associated with cascade completion. This supports similar findings in other studies [12, 14, 44]. Home-based and community-based HIV testing of young women have shown efficacy in facilitating early HIV diagnosis [20, 45]. Engagement in HIV care among women of childbearing age also improves PMTCT outcomes by reducing maternal viral load prior to the onset of pregnancy. This has been shown to minimize vertical transmission of HIV [46].

The significance noted for prior use of long-acting methods of contraception (including implants, injectables, and intrauterine devices) and cascade completion is interesting although this is likely a marker for health-seeking behavior or improved access to healthcare. This relationship persisted when adjusted for education and measures of household wealth. The negative association of cascade completion with ownership of a car, motorcycle, or bicycle is surprising but this may be explained by the distance from home to the healthcare facility or urban/rural residence (factors not captured in our analysis). In the univariate model, older age (>30) and larger household size (>3 persons) were associated with cascade completion but these did not persist in the adjusted model.

The strengths of this study include the large sample size with surveys administered at the community level in four African countries. This was devised to be more representative of the general population of women with a recent pregnancy. Study limitations include the fact that surveys were conducted in different settings in 2007–2009, an earlier era in terms of antiretroviral management and prevention of vertical transmission. There was less representation from Cameroon and Cote d'Ivoire in the study numbers. Since the currently recommended PMTCT regimen is daily, lifelong combination antiretroviral therapy (a more complex regimen compared to single dose nevirapine) and long-term engagement in HIV care are even more critical today than they were at the time of the survey. Many individual factors associated with cascade completion are expected to be similar over time among pregnant women in Africa. The underlying effects of stigma and HIV status disclosure with respect to the cascade were not assessed with the survey questionnaire.

## 5. Conclusions

Improved completion rates of the PMTCT cascade are necessary to reduce vertical HIV transmission in sub-Saharan Africa. Awareness of male partner HIV status, prepregnancy HIV diagnosis, and the use of long-term contraceptive methods were associated with cascade completion in this study. These may be markers for improved access to care and health literacy. Interventions targeting male partner testing and early HIV diagnosis should be evaluated to define their efficacy in increasing completion of the PMTCT cascade.

## Figures and Tables

**Table 1:** Baseline characteristics of survey respondents^*∗*^ (*n* = 976).

Variable	Completed PMTCT cascade (*n* = 355) *n* (%)	Did not complete PMTCT cascade (*n* = 621) *n* (%)	*p* value^*∗∗*^	Total *n* (%)
*Sociodemographics*				
Country of origin			<0.0001	
Cameroon	55 (15.4)	59 (9.5)		114 (11.7)
Cote d'Ivoire	1 (0.3)	52 (8.4)		53 (5.4)
South Africa	112 (31.6)	253 (40.7)		365 (37.4)
Zambia	187 (52.7)	257 (41.4)		444 (45.5)
Age			0.09	
<20	11 (3.1)	37 (6)		48 (4.9)
20–30	228 (64.2)	409 (65.9)		637 (65.3)
>30	116 (32.7)	175 (28.1)		291 (29.8)
Marital status			0.68	
Married	222 (62.7)	321 (52.2)		543 (56)
Living together	20 (5.7)	62 (10.1)		82 (8.5)
Divorced	10 (2.8)	10 (1.6)		20 (2)
Separated	17 (4.8)	42 (6.8)		59 (6.1)
Widowed	18 (5.1)	29 (4.7)		47 (4.9)
No partner	67 (18.9)	151 (24.6)		218 (22.5)
Education			0.56	
None/primary	157 (44.4)	270 (44)		427 (44.1)
Secondary or higher	197 (55.6)	344 (56)		541 (55.9)
Parity			0.08	
1	89 (25.8)	200 (33.4)		289 (30.6)
2	104 (30.1)	182 (30.5)		286 (30.3)
3	73 (21.2)	105 (17.6)		178 (18.9)
4	44 (12.8)	51 (8.5)		95 (10.1)
5+	35 (10.1)	60 (10)		95 (10.1)
Household size			0.05	
1-2	53 (15.1)	135 (22)		188 (19.5)
3	93 (26.5)	198 (32.3)		291 (30.2)
4	101 (28.8)	141 (23)		242 (25.1)
5+	104 (29.6)	139 (22.7)		243 (25.2)
Source of water			0.33	
Public tap	120 (33.8)	145 (23.4)		265 (27.2)
Protected well or piped	131 (36.9)	277 (44.6)		408 (41.8)
into plot				
Piped into house	63 (17.7)	120 (19.3)		183 (18.7)
Other	41 (11.6)	79 (12.7)		120 (12.3)
Own a television			0.97	
Yes	196 (55.8)	373 (60.6)		569 (58.8)
No	155 (44.2)	243 (39.4)		398 (41.2)
Own a cell phone			0.26	
Yes	252 (71.6)	440 (71)		692 (71.2)
No	100 (28.4)	180 (29)		280 (28.8)
Current employment Outside home			0.71	
Yes	142 (40.2)	225 (36.3)		367 (37.8)
No	211 (59.8)	394 (63.7)		605 (62.2)
Own a method of transportation			0.08	
Yes	77 (22.5)	156 (25.4)		233 (24.3)
No	266 (77.5)	459 (74.6)		725 (75.7)

*Health related*				
Timing of HIV diagnosis			<0.01	
Before pregnancy	43 (12.6)	7 (1.1)		50 (5.2)
During pregnancy	175 (51.2)	40 (6.6)		215 (22.5)
After pregnancy	124 (36.2)	565 (92.3)		689 (72.3)
Partner HIV testing			<0.01	
Negative	41 (11.6)	120 (19.5)		161 (16.6)
Positive	100 (28.3)	38 (6.2)		138 (14.2)
Not tested/do not know	212 (60.1)	458 (74.3)		670 (69.2)
Use of birth control (ever)			<0.01	
Modern method				
Injectables/implants	119 (33.7)	156 (25.3)		275 (28.4)
Pill	68 (19.3)	123 (19.9)		191 (19.7)
Condoms	37 (10.5)	26 (4.2)		63 (6.5)
M/F sterilization or	2 (0.6)	4 (0.7)		6 (0.6)
hysterectomy				
Intrauterine device	1 (0.3)	3 (0.5)		4 (0.4)
Lactational	1 (0.3)	2 (0.3)		3 (0.3)
amenorrhea				
Traditional or no				
method				
No method	125 (35.3)	298 (48.3)		423 (43.6)
NFP or withdrawal	0 (0)	5 (0.8)		5 (0.5)
Timing of ANC entry			0.29	
1st trimester	56 (15.8)	129 (21.7)		185 (19.5)
2nd trimester	254 (71.6)	390 (65.8)		644 (67.9)
3rd trimester	45 (12.6)	74 (12.5)		119 (12.6)
ANC location			<0.01	
Hospital	51 (14.4)	88 (14.2)		139 (14.2)
Health center	291 (82)	460 (77)		751 (77)
Health post	8 (2.3)	22 (3.1)		30 (3.1)
Other	5 (1.4)	51 (5.7)		56 (5.7)
ANC facility type			<0.01	
Governmental	312 (87.9)	529 (85.2)		841 (86.2)
Faith based organization	38 (10.7)	41 (6.6)		79 (8.1)
Other	5 (1.4)	51 (8.2)		56 (5.7)

^*∗*^Missing values: marital status 7, education 8, parity 33, household size 12, own a television 9, own a cell phone 4, current employment outside home 4, own a method of transportation 18, timing of HIV diagnosis 22, partner HIV testing 7, use of birth control (ever) 6, and timing of ANC entry 28.

^*∗∗*^ *p* values calculated from univariate logistic regression models which included site as a stratification factor. The *p* value for family planning compares modern methods to traditional/no method.

PMTCT: prevention of mother to child HIV transmission, ANC: antenatal clinic, and NFP: natural family planning.

**Table 2:** Factors associated with cascade completion in the PEARL study^*∗∗*^.

Variable	Univariate analysis OR (95% CI)	Multivariable analysis OR (95% CI) *n* = 843
Maternal age (years)		
<20	Ref	Ref
20–30	1.8 (0.9–3.7)	1.8 (0.8–4.2)
>30	2.2 (1.1–4.5)^*∗*^	1.9 (0.8–4.5)
Marital status		
No partner	Ref	Ref
Partner	1.1 (0.8–1.6)	0.8 (0.5–1.3)
Education		
None/primary	Ref	Ref
Secondary or higher	1.1 (0.8–1.5)	1.1 (0.8–1.5)
Parity		
1	Ref	Ref
2	1.3 (0.9–1.8)	0.8 (0.5–1.4)
3	1.4 (0.9–2.1)	0.7 (0.3–1.4)
4	1.9 (1.2–3.2)^*∗*^	1 (0.4–2.5)
5+	1 (0.6–1.7)	0.5 (0.2–1.4)
Household size		
1-2	Ref	Ref
3	1.2 (0.8–1.8)	1.4 (0.7–2.7)
4	1.7 (1.1–2.5)^*∗*^	2.3 (1.0–5.4)
5+	1.6 (1.04–2.4)^*∗*^	2.3 (0.8–6.6)
Source of water		
Protected well or piped into plot	Ref	N/A
Piped into house	1.1 (0.7–1.6)	
Public tap	1.2 (0.9–1.8)	
Other	0.8 (0.5–1.3)	
Own a television		
No	Ref	N/A
Yes	1 (0.8–1.3)	
Own a cellphone		
No	Ref	Ref
Yes	1.2 (0.9–1.6)	1.3 (0.9–1.9)
Employment		
No	Ref	N/A
Yes	1 (0.7–1.3)	
Own a method of transportation		
No	Ref	Ref
Yes	0.8 (0.5–1)	0.7 (0.5–0.98)^*∗*^
Timing of HIV diagnosis		
During/after pregnancy	Ref	Ref
Before pregnancy	12.3 (5.3–28.6)^*∗*^	14.1 (5.2–38.6)^*∗*^
Partner HIV status		
Unknown	Ref	Ref
Known	1.7 (1.3–2.2)^*∗*^	1.4 (1.01–2.0)^*∗*^
Family planning		
Traditional/none	Ref	Ref
Short acting modern method	1.3 (1–1.9)	1.3 (0.8–1.9)
Long acting modern method	2.3 (1.6–3.2)^*∗*^	2.0 (1.3–3.0)^*∗*^
Timing of ANC entry		
1st trimester	Ref	Ref
2nd trimester	1.3 (0.9–1.9)	1.3 (0.9–2.0)
3rd trimester	1.4 (0.9–2.3)	1.3 (0.7–2.2)
ANC location		
Health center	Ref	N/A
Hospital	1.1 (0.7–1.6)	
Health post	1.2 (0.4–3.5)	
Other	0.2 (0.1–0.5)^*∗*^	
ANC facility type		
Governmental	Ref	Ref
Faith based organization	1.2 (0.7–1.9)	1.2 (0.7–2.1)
Other	0.2 (0.1–0.5)^*∗*^	0.3 (0.1–0.9)^*∗*^

^*∗*^ *p* value < 0.05.

^*∗∗*^Logistic regression models. All models include site as a stratification factor. The adjusted model includes all of the variables in the table with odds ratios listed. Data for variables that were not included in the multivariable model are labeled “N/A.”
